# Fermented *Rosa Roxburghii* Tratt Juice Alleviates High-Fat Diet-Induced Hyperlipidemia in Rats by Modulating Gut Microbiota and Metabolites

**DOI:** 10.3389/fphar.2022.883629

**Published:** 2022-05-20

**Authors:** Jiacheng Ji, Shuo Zhang, Minyan Yuan, Min Zhang, Li Tang, Pengjiao Wang, Yujie Liu, Changqian Xu, Peng Luo, Xiuli Gao

**Affiliations:** ^1^ State Key Laboratory of Functions and Applications of Medicinal Plants & School of Pharmacy, Guizhou Medical University, Guiyang, China; ^2^ Microbiology and Biochemical Pharmaceutical Engineering Research Center of Guizhou Provincial Department of Education, Guizhou Medical University, Guiyang, China; ^3^ Experimental Animal Center of Guizhou Medical University, Guiyang, China; ^4^ Guizhou Provincial Engineering Research Center of Food Nutrition and Health, Guizhou Medical University, Guiyang, China

**Keywords:** fermented rosa roxburghii tratt juice, gut microbiota, metabolomic, hyperlipidemia, lipid metabolism

## Abstract

Hyperlipidemia endangers human health and has become a significant public health problem. This study aimed to investigate the mechanism of the hypolipidemic effects of Fermented *Rosa roxburghii* Tratt juice (FRRT) on hyperlipidemic rats and a new hypolipidemic intervention strategy was disclosed. The study revealed 12 weeks FRRT treatment significantly decreased the body weight, total cholesterol (TC), triacylglycerol (TG), low-density lipoprotein cholesterol (LDL-c), while high-density lipoprotein cholesterol (HDL-c) increased. We integrated the 16S rDNA sequencing and metabolomic profiling to evaluate the changes in the gut microbiota and metabolites. Significant changes in microbial composition accompanied marked changes in 56 feces metabolites. The results showed that FRRT could decrease the ratio of *Firmicutes* to *Bacteroidetes*, while increase the abundance of some bacterial genera (*Prevotella*, *Paraprevotellaceae_Prevotella*, *Ruminococcus*, *Oscillospira*). Metabolomics analysis displayed that the metabolisms of bile acid, amino acid and lipid were significantly affected by FRRT. Correlation analysis suggest that the reductions in serum lipids by FRRT are associated with the gut microbial community and their associated metabolites (amino acid metabolites, bile acid metabolites, and lipid metabolites). This study confirmed FRRT could be used as a new dietary and therapeutic strategy to dyslipidemia by improving the gut microbiota dysbiosis, metabolomic disorders and regulating the dyslipidemia. Our study also extended the understanding of the relationship between gut microbiota, metabolites, and lipid-lowering functions.

## Introduction

Hyperlipidemia is a systemic disorder of lipid metabolism, including elevated levels of total cholesterol (TC), triglycerides (TG), low-density lipoprotein cholesterol (LDL-C) and a decreased level of high-density lipoprotein cholesterol (HDL-C). Hyperlipidemia can lead to a series of cardiovascular diseases such as coronary heart disease, cerebral thrombosis, and atherosclerosis (Abliz et al., 2014; Chen et al., 2014; Pirillo et al., 2021). With the improvement of living conditions, the excessive intake of high-fat diets, and the lack of exercise, results in more and more lipids accumulated in the body and the thus leading to Hyperlipidemia (Pirillo et al., 2021). To prevent and treat cardiovascular and cerebrovascular diseases could be by way of regulating lipid metabolism and reduce lipid levels. Therefore, it is important to explore new and effective intervention strategies for hyperlipidemia.

Studies have shown that gut microbiota can regulate the metabolism of the host of cholesterol and lipid, which is closely related to hyperlipidemia (Deng et al., 2018; Jin et al., 2018). The high-fat diet can affect the microenvironment of gut microbiota that significantly decrease the abundance of Bacteroides, while significantly increase the abundance of Firmicutes (Nguyen et al., 2017; Chen et al., 2019), leading to gut microbiota dysbiosis. The dysbiosis further disrupts lipid metabolism. which can form a vicious cycle and then causes hyperlipidemia. Some studies have shown that increasing the abundance of some beneficial bacteria could improve hyperlipidemia. Therefore, gut microbiota dysbiosis is closely related to hyperlipidemia, and gut microbiota might be a new target for the treatment of hyperlipidemia.

Bile acids (BAs) are the dominant downstream products of cholesterol catabolism, and include primary and secondary bile acids. Many studies point out that a high-fat diet may show a positive correlation with the production of secondary bile acids such as deoxycholic acid (DCA) and lithocholic acid (LCA), and evidence indicates that DCA and LCA are toxic bile acids associated with metabolic syndrome, and gastrointestinal diseases (Bernstein et al., 2006; Stenman et al., 2012; Stenman et al., 2013). Therefore, the production and excretion of BAs are critical for the maintenance of cholesterol homeostasis.

*Rosa roxburghii* Tratt (RRT), a medicinal plant and traditional food in southwest China, and included in the Quality Standards for Chinese and Ethnic Medicinal Herbs of Guizhou Province as ethnic medicinal herbs of Guizhou. RRT riches in organic acid, vitamin C, superoxide dismutase, flavonoids, polyphenols, polysaccharides and other active ingredients (Liu et al., 2016; Wang et al., 2021). In recent years, some studies have pointed out that *Rosa roxburghii* Tratt juice (RRTJ) has anti-tumour, anti-atherogenic, anti-oxidant, and anti-hyperlipidemic effects (Xu et al., 2019; Wang et al., 2021; Li et al., 2022). RRTJ also can affect the expression of proteins related to lipid metabolism to maintain metabolic disorders (Zhang et al., 2001; Lu and Bao, 2002; Song and Shen, 2020). Moreover, the extracted water-soluble polysaccharides from RRT fruit (RTFP) showed inhibitory activities against *a*-glucosidase and the potential to lower blood glucose, which also could reverse diabetes-induced gut microbiota dysbiosis (Huizhu Wang et al., 2018; Lei Wang et al., 2018; Hao Wang et al., 2019). The total polyphenols (PFs) from *Rosa roxburghii* Tratt showed modulating effects on hyperlipidemia and oxidative stress (Zhao et al., 2016; Wei et al., 2017; Lei Wang et al., 2019).

RRT contains large number of bioactive ingredients, while fresh RRT fruit is not suitable for long-term storage at room temperature. Processed RRT products by fermentation could maximize the preservation of active ingredients, and improve the taste and the flavor. At present, the mechanism of action of FRRT on hyper-lipidemia has not been explored in depth. Therefore, in this paper, we investigated the effect of FRRT on high-fat diet-induced hyperlipidemia, and unraveled the mechanism of action at the level of gut microbiota and metabolites in rats, to provide a deeper theory for FRRT as a new therapeutic strategy to prevent hyperlipidemia.

## Materials and Methods

### Preparation and Determination of FRRT

The FRRT (Batch Number:200715) was obtained from Guizhou Longshan Xiangfang Food Co., Ltd (Guizhou, China). The Angel active yeast BV818 was obtained from Angel Yeast Co., Ltd (Hubei, China). The fresh RRT fruit of “Guinong No.5” was selected and identified by the Key Laboratory of Natural Product Chemistry of the Chinese Academy of Sciences (Guizhou, China). FFRT was prepared as follows. After selection and cleaning, fresh RRT fruit was crushed and pressed to get RRT juice, and the RRTJ juice was filtered and reserved. Then the activated pectinase was added to RRT juice and macerated at 10°C for 16 h, and filtered. The filtered RRT juice was transferred to a temperature-controlled fermenter. The crystal sugar was added to adjust the sugar level to 200 g/L, inoculated with activated Angel active yeast BV818 200 mg/L, and fermented for 3 months. At last, FRRT was obtained by sterilization and filtration.

The contents of total polyphenols, total flavonoids, total polysaccharides were measured by UV spectrophotometry (Shimadzu, Japan), and the contents of the vitamin C were used by HPLC (Agilent, United States) (Tan et al., 2019; Guan et al., 2020; Lian et al., 2021).

### Experimental Animals

Male Sprague-Dawley rats were obtained from the Laboratory Animal Center of Guizhou Medical University (Permit Number: SYXK Qian 2018-0001) and housed in an animal room (temperature: 23 ± 2°C; relative humidity = 60 ± 5%; 12 h light/12 h dark cycle). All animal experiments were performed according to protocols approved by the experimental animal ethics committee of Guizhou Medical University.

After 1 week of acclimation, twelve rats were fed a high-fat diet for 8 weeks to induce hyperlipidemia. Then, Rats were randomly divided into two groups (*n* = 6/group): high-fat diet (HFD) group and high-fat diet + FRRT (HFD + FRRT) group. In our previous experiments, we set up two different dose groups (5 ml/kg/day and 10 ml/kg/day), and we found that the treatment effect was better at the dose of 10 ml/kg/day. We chose the dose of 10 ml/kg/day for further experimental analysis (Supplementary Table S1). The HFD group was fed a high-fat diet for 12 weeks, and the HFD + FRRT group was fed a high-fat diet and gavaged with FRRT (10 ml/kg/day) for 12 weeks. The high-fat diet contains 79.8% basal feed, 18% lard, 0.2% sodium cholate, 2% cholesterol.

At the end of the treatment period, blood, feces, and Liver tissues were collected and processed for biochemical analysis, histological analysis, 16S rRNA gene sequence, and targeted metabolomics.

### Biochemical Analysis

The blood samples were kept at room temperature for 1 hour and centrifuged at 10 min, 3000 rpm/min. The serum fraction was separated from each sample. Finally, the levels of triglyceride (TG), total cholesterol (TC), low-density lipoprotein cholesterol (LDL-c), high-density lipoprotein cholesterol (HDL-c), alanine aminotransferase (ALT), and aspartate aminotransferase (AST) in serum were measured by using commercial kits (Nanjing Jiancheng Bioengineering Institute, Nanjing, China).

### Hematoxylin-Eosin Staining

The liver tissue was fixed in 10% paraformaldehyde for 24 h, embedded in paraffin, cut into 4-µm sections, and followed by hematoxylin-eosin (H&E) staining.

### Gut Microbiota Analysis

The total bacterial DNA of each fecal sample was extracted from the fecal samples using OMEGA Soil DNA Kit (Omega Bio-Tek, Norcross, GA, United States). PCR amplification of the V3-V4 regions of 16S rRNA genes was performed using the bacterial primers 338F (5′-ACT​CCT​ACG​GGA​GGC​AGC​A-3′) and 806R (5′-GGACTACHVGGGTWTCTAAT-3′), and sequencing was performed using the Illumina NovaSeq platform. Sequences were then quality filtered, denoised, merged and chimera removed using the DADA2 plugin, Sequence data analyses were mainly performed using QIIME2 (https://docs.qiime2.org/2019.4/tutorials/) and R packages (v3.2.0).

### Metabolic Profiling Analysis

Targeted metabolomic analysis of stool samples was performed by Metabolo-Profile (Shanghai, China). Sample preparation and quantitative measurement were based on previously published methods (Xie et al., 2021). Briefly, 5 mg of lyophilized feces were homogenized with zirconium oxide beads, 25 µL of deionized water and 120 µL of methanol containing internal standard to extract the metabolites and then the supernatant was removed by centrifugation. The resulting supernatants were subjected to derivatization with 3-nitrophenylhydrazine (3-NPH) and N-(3-(dimethylamino)propyl)-N′-ethylcar-bodiimide (EDC)·HCl. Subsequently, the derivatized samples were analyzed by ultra-performance liquid chromatography coupled to a tandem mass spectrometry (UPLC-MS/MS) system (ACQUITY UPLC-Xevo TQ-S, Waters Corp, Milford, MA, United States). All of the standards were purchased from Sigma-Aldrich (St. Louis, MO, United States), Steraloids Inc (Newport, RI, United States) and TRC Chemicals (Toronto, ON, Canada).

### Statistical Analysis

Significant differences between the two groups were analyzed by Student’s unpaired *t*-test using SPSS 23.0, and *p* < 0.05 was considered statistically significant. Raw fecal metabolome data files generated by UPLC-MS/MS were processed by Targeted Metabolome Batch Quantification (TMBQ) software (V1.0, HMI, Shenzhen, Guangdong, China). Pareto-scaled principal component analysis (PCA) and orthogonal partial least-squares discriminant analysis (OPLS-DA) were subjected to multivariate data analysis. OPLS-DA, PCA, and Z-Score heat map were analyzed by iMAP (V1.0, MetabO-Profile, Shanghai, China) platform.

## Results and DISCUSSION

### Chemical Composition of FRRT

The contents of total flavonoids were 13.49 ± 0.38 mg/mL, the total polysaccharides were 23.91 ± 0.26 mg/mL, the total polyphenols were 35.97 ± 0.41 mg/mL, and the vitamin C were 7.58 ± 0.10 mg/mL.

### FRRT Improves Dyslipidemia in Hyperlipidemic Rats

**FIGURE 1:**
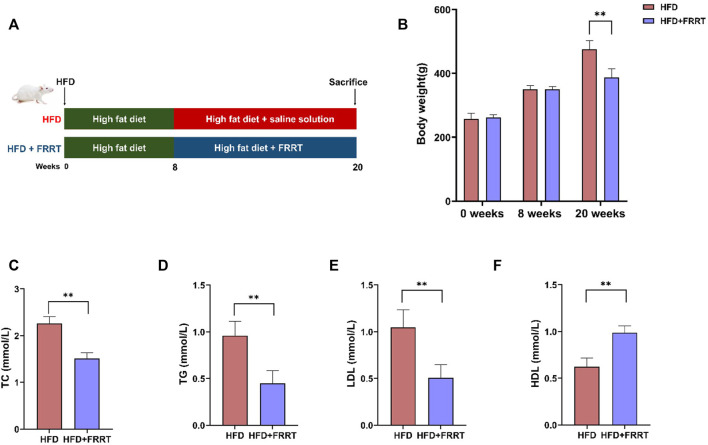
FRRT improves dyslipidemia in HFD-induced hyperlipidemic Rats. **(A)** Schematic diagram of FRRT treatment. **(B)** Rats’ body weight at weeks 0, 8, and 20. Serum TC **(C)**, TG **(D)**, LDL-c **(E)**, and HDL-c **(F)** levels were measured using commercial kits. Data are presented as mean ± SEM (*n* = 6). *p < 0.05, **p < 0.01 compared with HFD group.

The period of the experiment is 20 weeks, and the animal experiment procedure is shown in Figure 1A. At the end of the treatment, the bodyweight of rats with FRRT treatment significantly decreased compared with the HFD group (Figure 1B, *p* < 0.01). At the same time, rats with FRRT treatment exhibited lower levels of TC, TG, LDL-c, and a higher level of HDL-c compared with HFD rats (Figure 1C–F, *p* < 0.01), suggesting that FRRT could effectively ameliorate dyslipidemia in hyperlipidemia rats.

### FRRT Improves Liver Function Damage in Hyperlipidemic Rats

**FIGURE 2:**
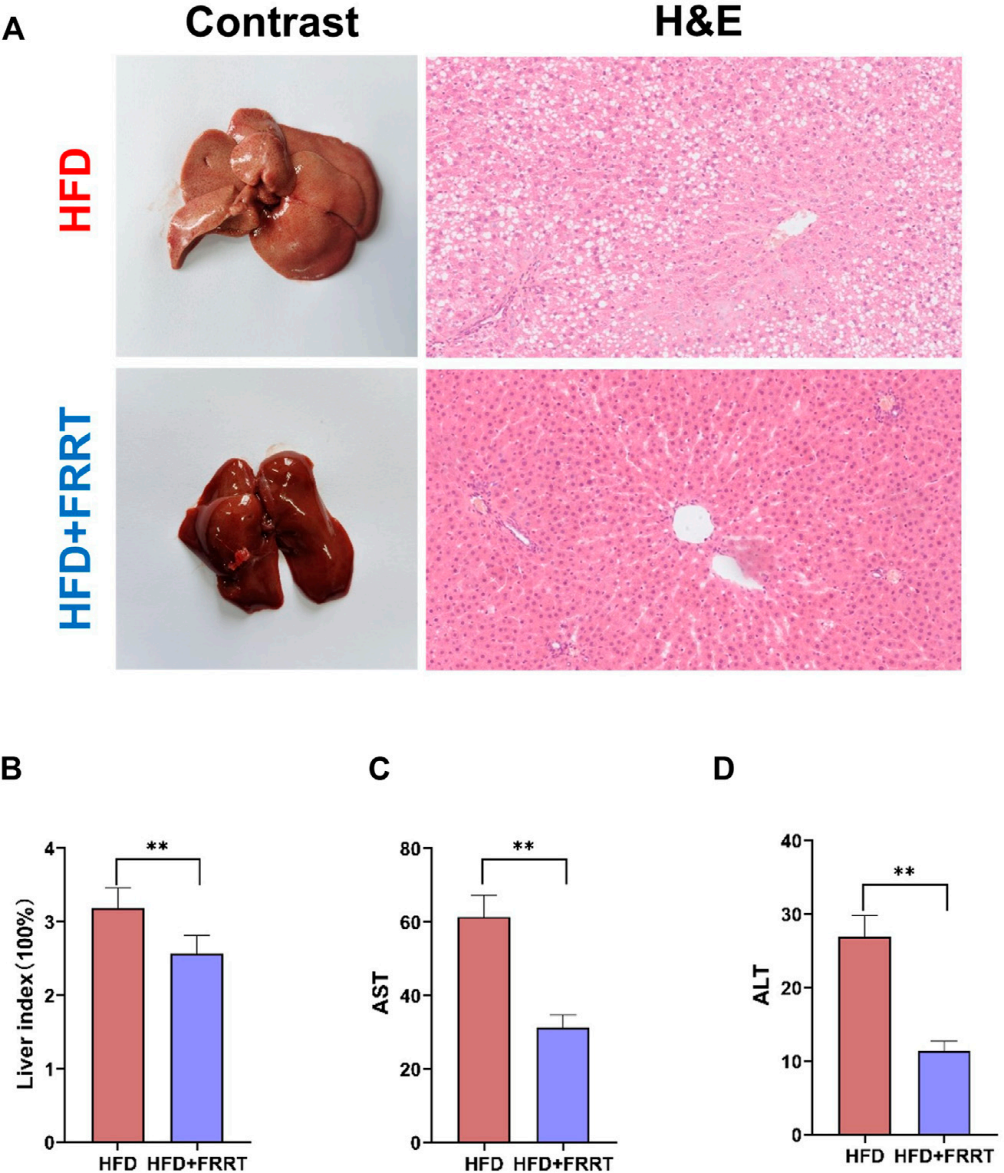
FRRT improves liver function damage in hyperlipidemic rats. **(A)** H&E staining images and morphology of Liver. **(B)** Changes in the liver index. Serum AST **(C)** and ALT **(D)** levels were measured using commercial kits. Data are presented as mean ± SEM (*n* = 6). *p < 0.05, **p < 0.01 compared with HFD group.

We further analyzed the liver function damage in the HFD-induced hyperlipidemic Rats. Histological analysis showed that the structure of the hepatic lobule in hyperlipidemic rats is severely disturbed, the fat vacuoles occupy less than 3/4 of the entire field of view, and the fat vacuoles are fused to form a sheet. Some of the hepatocytes in the area where the fat vacuoles are concentrated are enlarged, with light cytoplasmic colorin. Some of the cells are necrotic and the nuclei are dissolved. Moreover, the volume and number of lipid accumulation and fat vacuoles in the liver were significantly reduced after FRRT treatment. The hepatocytes were regularly arranged with prominent nuclei and ribosomes and well-defined cytoplasm (Figure 2A). The liver index reflects the extent of fat deposition in the liver to some extent. After FRRT treatment, the liver index was significantly reduced in the HFD group (Figure 2B, *p* < 0.01). In addition, it also reduced the levels of AST and ALT (Figures 2B–D, *p* < 0.01), indicating that FRRT improved the liver function impairment caused by HFD.

### FRRT Ameliorates the Gut Microbial Dysbiosis in HFD-Induced Hyperlipidemic Rats

**FIGURE 3:**
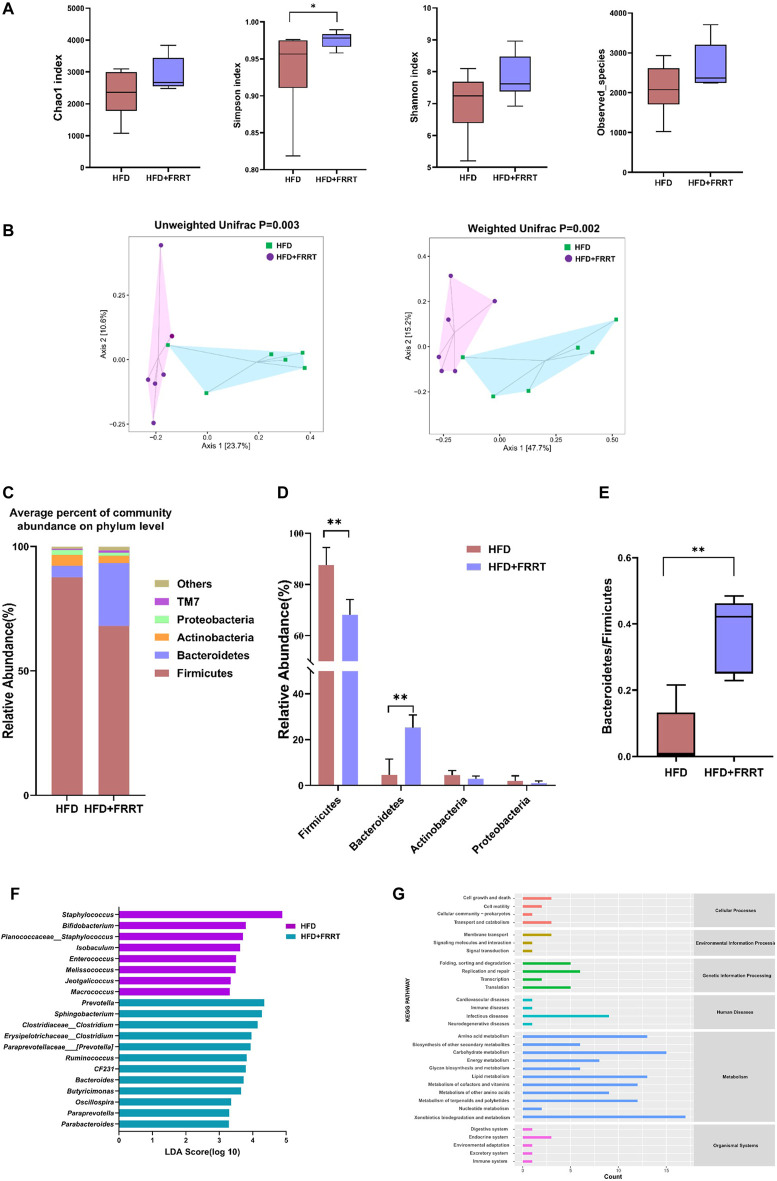
The alteration of the gut microbiome in HFD rats. **(A)** The *a*-diversity indexes of Chao1, Shannon, Simpson, and observed-species of gut microbiota between HFD and HFD + FRRT rats. **(B)** Unweighted based PCoA and weighted UniFrac based PCoA between gut bacterial communities of HFD and HFD + FRRT rats. *p* values were determined by the ADONIS test. Differential abundances of genera were determined by the Wilcoxon rank-sum test and Mann-Whitney *U* test. **(C)** The average percent of community abundance on phylum level in HFD and HFD + FRRT groups. **(D)** Relative abundance of Firmicutes, Bacteroidetes, Actinobacteria, Proteobacteria, and the Bacteroidetes/Firmicutes abundance ratio **(E)** in fecal microbiota between HFD and HFD + FRRT. **(F)** The LEfSe analysis of the gut microbiota differed between HFD and HFD + FRRT groups. **(G)** The effect of the gut microbiota modifications on predicted functional metabolic pathways obtained from PICRUSt analysis of 16S rDNA sequencing data.

To investigate the effects on gut microbiota in HFD-induced hyperlipidemia rats given FFRT, we performed 16S rDNA high-throughput sequencing on rat feces. We firstly compared the microbiota diversity between the HFD and HFD + FRRT (Figure 3A). The richness and diversity of the bacterial community (Chao1, Simpson, Shannon, and observed species) were increased after FRRT treatment. Additionally, a significant difference was observed in *ß*-diversity based on the un-weighted (*p* = 0.002) and weighted (*p* = 0.003) UniFrac between the HFD and HFD + FRRT groups (Figure 3B). These results suggest that FRRT has a significant impact on intestinal microbiota composition.

Furthermore, we analyzed the effect of FRRT on intestinal microbiota at the phylum and genus levels. At the phylum levels, *Firmicutes*, *Bacteroides*, *Proteobacteria*, and *Actinobacteria*, were the dominant phyla, accounting for more than 85% of the relative abundance (Figure 3C). The FRRT treatment significantly lower the abundance of *Firmicutes* and increase the abundance of *Bacteroides*, and yielded a significantly (*p* < 0.05) increased *Bacteroidetes* to *Firmicutes* ratio (Figures 3D,E). At the genus levels, LEfSe analysis showed that the HFD rats had a higher abundance of *Staphylococcus*, *Bifidobacterium*, *Isobaculum*, *Enterococcus*, *Melissococcus*, *Jeotgalicoccus*, *Macrococcus*, whereas the HFD + FRRT rats primarily showed higher enrichment of *Prevotella*, *Sphingobacterium*, *Clostridium*, *Erysipelotrichaceae_Clostridium*, *Paraprevotellaceae_Prevotella*, *Ruminococcus*, *CF231*, *Bacteroides*, *Butyricimonas*, *Oscillospira*, *Paraprevotella*, *Parabacteroides* (Figure 3F).

PICRUSt analysis showed that the gut microbiota was predicted to be related to various metabolic functions, such as amino acid metabolism, energy metabolism, lipid metabolism, and carbohydrate metabolism (Figure 3G).

In brief, FRRT significantly affected the composition of gut microbiota in HFD-induced hyperlipidemic rats, and these altered gut microbiotas were closely related to the function of the lipid metabolism and amino acid metabolism in rats.

### Alteration of the Fecal Metabolome in HFD-Induced Hyperlipidemic Rats

**FIGURE 4:**
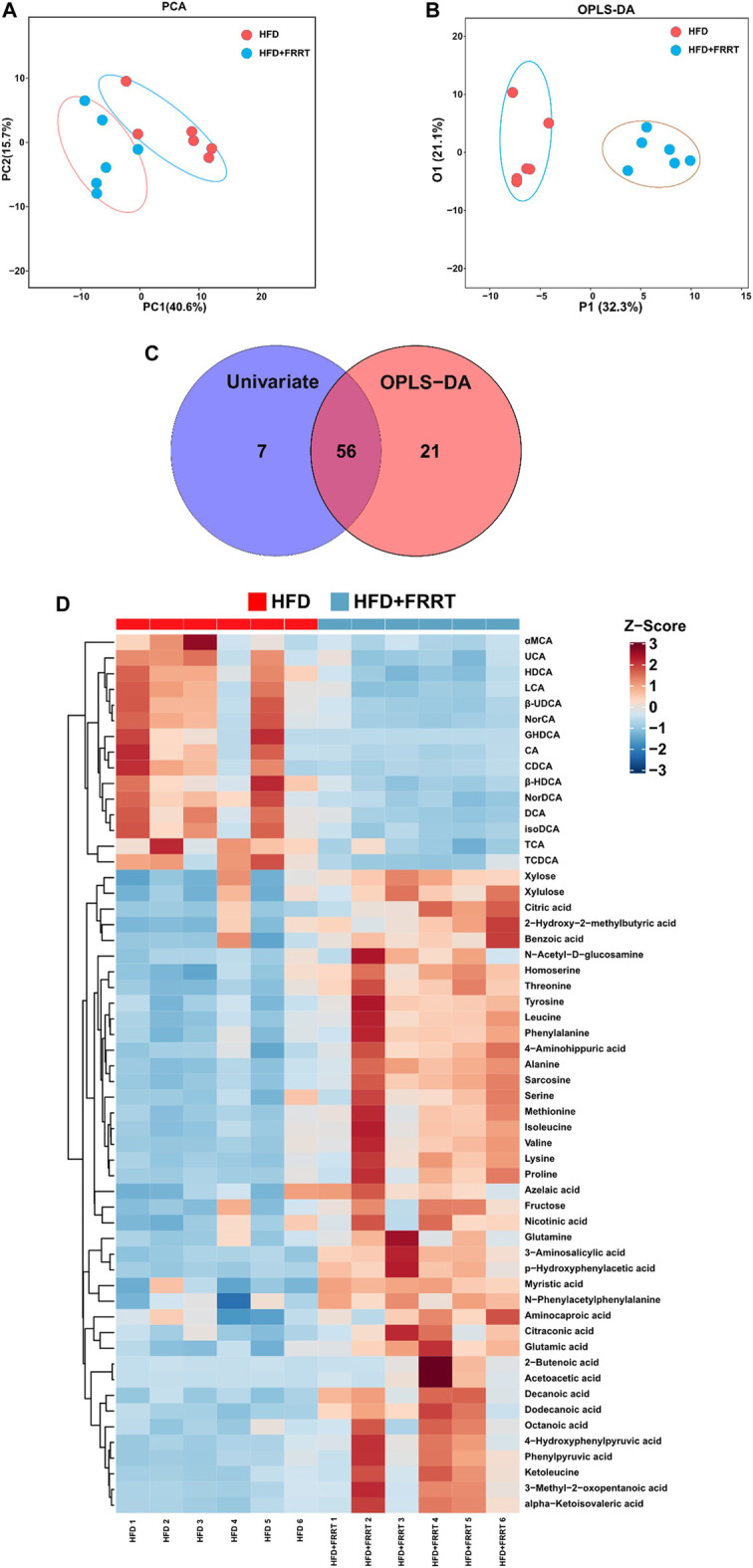
Metabolic profile as well as multivariate/Univariate analysis and metabolic pathway of metabolites between HFD and HFD + FRRT group. **(A)** PCA plots with the scores of the first two principal components based on 160 metabolites. **(B)** OPLS-DA plots with the scores of the first two principal components. **(C)** Venn Plot of differential metabolites, by getting union/intersection of the differential metabolites from univariate statistics and multi-dimensional statistics. **(D)** Heatmap analysis of potential biomarkers, the concentration value is converted to Z-score by standardized Z-score transformation.

Metabolites produced by gut microbiota fermentation may influence host metabolism. A targeted metabolic profile analysis of 160 metabolites was conducted to study the differential metabolites in the feces. Score plots of PCA and OPLS-DA showed that the levels of fecal metabolites changed significantly after FRRT treatment (Figures 4A,B). Furthermore, by getting the intersection of the differential metabolites from univariate statistics and multi-dimensional statistics, we find 56 potential biomarkers which may play critical roles in biological processes (Figure 4C). Also, as shown in Figure 4D, these metabolites were mainly involved in amino acid metabolism (phenylalanine and tyrosine metabolism, valine, leucine and isoleucine degradation, glycine and serine metabolism. etc.), fatty acid metabolism (beta-oxidation of very-long-chain fatty acids, dodecanoic acid, myristic acid, citraconic acid. etc.), bile acids metabolism (HDCA, LCA, DCA, etc.). In brief, HFD was closely associated with altered amino acids metabolisms, bile acids metabolisms, and lipid metabolisms.

### Association Between Gut Microbial, Metabolites, and Metabolic Parameters

**FIGURE 5:**
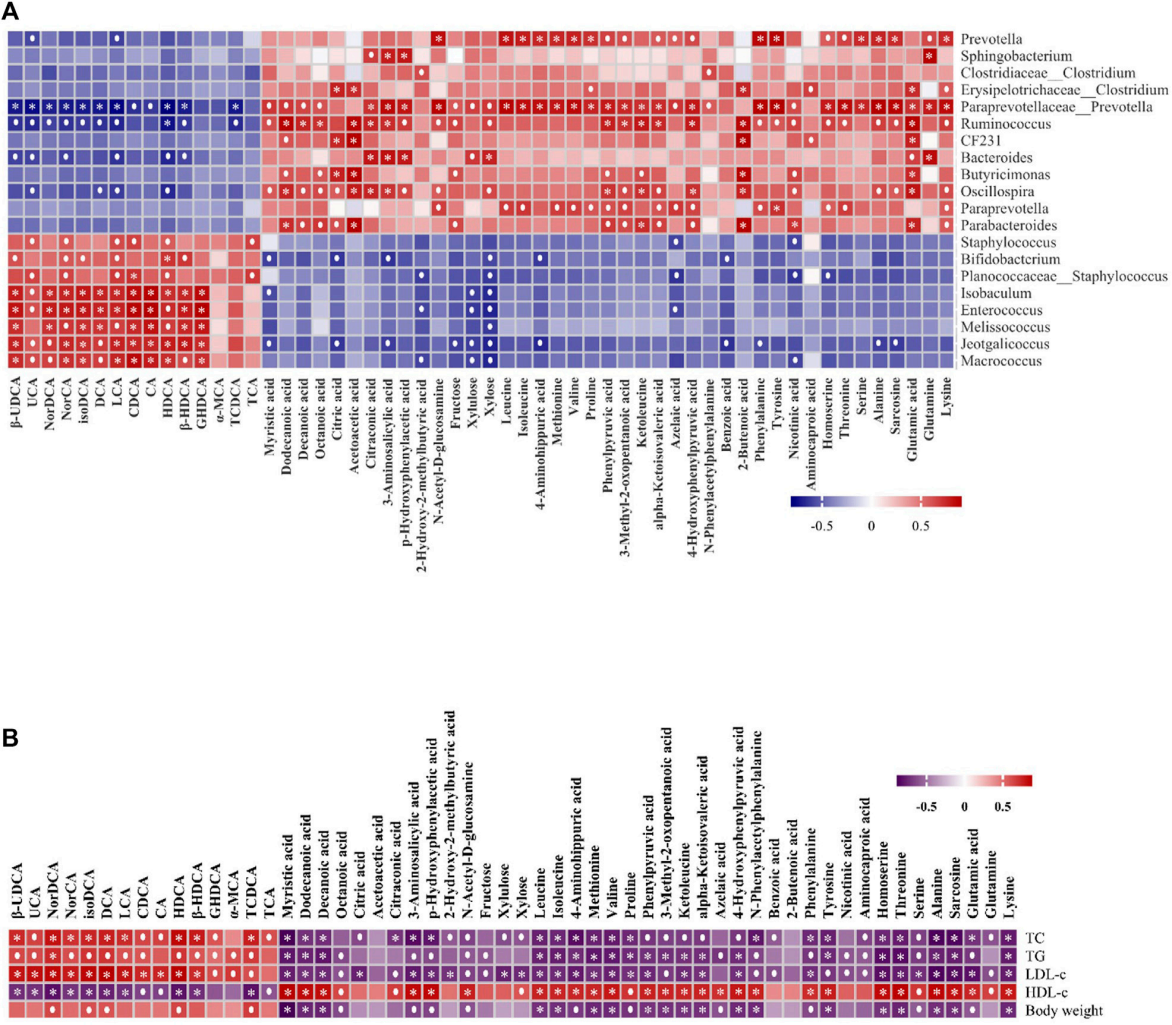
The Spearman’s correlation analysis of gut microbiota, metabolic parameters, and metabolites. **(A)** Correlations of 56 altered metabolites and five metabolic parameters in HFD and HFD + FRRT using Spearman correlation analysis. **(B)** Correlations of 56 altered metabolites and 20 altered gut microbiota in HFD and HFD + FRRT using Spearman correlation analysis.

To further investigate the linkages between gut microbiota, metabolites, and metabolic parameters and their regulatory effects, we used Spearman’s rank correlation analysis to analyze the correlation between the 56 altered metabolites and 20 altered gut microbiota in the HFD and the HFD + FRRT group. The altered metabolites were mainly involved in amino acid metabolites (glutamine, glutamic acid, alanine, etc.), fatty acid metabolites (dodecanoic acid, myristic acid, decanoic acid, etc.), bile acids metabolites (HDCA, LCA, DCA, etc.). As shown in Figure 5A, the altered metabolites were significantly associated with *Prevotella*, *Paraprevotellaceae*_*Prevotella*, *Ruminococcus*, *Oscillospira*, *Staphylococcus*, *Enterococcus*, and *Macrococcus*.

We also performed Spearman’s rank correlation analysis of 56 altered metabolites with five metabolic parameters. The result suggested that the altered metabolites of amino acid metabolites (glutamine, glutamic acid, alanine, etc.), fatty acid metabolites (dodecanoic acid, myristic acid, decanoic acid, etc.), bile acids metabolites (HDCA, LCA, DCA, etc.) were significantly associated with blood lipid levels and bodyweight Figure 5B. The results suggested that FRRT facilitated its antihyperlipidemic effect by regulating the composition of gut microbiota and its metabolites.

## Discussion

Hyperlipidemia is one of the components of metabolic syndrome, of which the pathophysiology is very complex and has been only partially elucidated. In this study, FRRT treatment can effectively improve the body weight and lipid levels in rats with high-fat diet-induced hyperlipidemia. Meanwhile, FRRT improved liver function impairment and mainly exerts a hypolipidemic effect by regulating the composition of gut microbiota and its metabolites.

The gut microbiota acts as an independent and stable ecosystem in the body. It carries out various metabolic activities in the human body and produces a series of metabolites that affect the health of the host (Fan and Pedersen, 2021). In recent decades, the gut microbiota, dominated by Firmicutes and Bacteroidetes, played an important role in the development of obesity (Turnbaugh et al., 2006; Murphy et al., 2010). Many studies demonstrated that a higher abundance ratio of *Firmicutes* to *Bacteroidetes* is positively correlated to obesity in obese mice and humans (Nguyen et al., 2017; Xiao et al., 2017; Zhao et al., 2017). Compared with the healthy rats in reported studies, the abundance of *Firmicutes* increased, and the abundance of *Bacteroidetes* decreased at the phylum in the HFD group (Chen et al., 2019). In this study, the abundance of *Firmicutes* was significantly decreased, and the abundance of *Bacteroides* was significantly increased in HFD + FRRT treatment. The ratio of *Firmicutes* to *Bacteroidetes* was also correspondingly reduced after FRRT treatment.

To further assess specific changes in the gut microbiota, we compared the relative abundance at the genus level between HFD and FRRT + HFD groups. We compared the relative abundance at the genus level between HFD and healthy rats, the relative abundances of *Staphylococcus* in the HFD group increased, whereas those of *Oscillospira*, *Prevotella*, *Ruminococcus* and *Clostridium* decreased compared with the healthy rats in the reported studies (Chen et al., 2019). In the current study, the FRRT significantly decreased the abundance of *Staphylococcus*, *Bifidobacterium*, *Isobaculum*, *Enterococcus*, *Melissococcus*, *Jeotgalicoccus*, *Macrococcus*, and increased the abundance of *Prevotella*, *Sphingobacterium*, *Clostridium*, *Erysipelotrichaceae_Clostridium*, *Paraprevotellaceae_Prevotella*, *Ruminococcus*, *CF231*, *Bacteroides*, *Butyricimonas*, *Oscillospira*, *Para-prevotella*, *Parabacteroides*. Among them, the reduction in *Staphylococcus* (LDA = 4.88, *p* = 0.037) and the increase in *Prevotella* (LDA = 3.94, *p* = 0.0033) were the most significant.

Our results showed the abundance of *Staphylococcus* enriched in high-fat diet-induced hyperlipidemia rats, and the relative abundance of *Staphylococcus* was dramatically decreased after FRRT treatment. Moreover, *Staphylococcus* is opportunistic pathogen genera (Foster, 2005; Wertheim et al., 2005; Jenkins et al., 2015), which can cause several types of infections, and colonization has been reported to occur easier in obese individuals and patients (van Belkum et al., 2009; Olsen et al., 2013). Besides, Enterococcus has been reported to promote intestinal inflammation by compromising the integrity of the epithelial barrier (Steck et al., 2011; Kao and Kline, 2019). We also observed the same phenomenon, but both of these microorganisms were suppressed by FRRT.

*Prevotella*, a dominant gut bacterium belongs to the phylum *Bacteroidetes*. Nutritional interventions with fiber-rich foods usually increase *Prevotella* abundance (Haro et al., 2017; Benítez-Páez et al., 2019; Roager et al., 2019; Ghosh et al., 2020). Increased *Prevotella* in gut microbiota has been reported to increase weight loss, lower cholesterol levels (Eriksen et al., 2020), and limit the action of *bifidobacterial* (Chung et al., 2020), which may reveal the phenomenon of the abundance of *bifidobacteria* decreased after FRRT treatment. However, we need further experiments to verify this phenomenon. Besides, *Oscillospira* abundance was also strongly associated with obesity, leanness, and human health (Tims et al., 2013; Goodrich et al., 2014; Cao et al., 2020). Numerous evidence suggest that the *Oscillospira* abundance plays an important role in the metabolic activities associated with hyperlipidemia and obesity (Escobar et al., 2014; Del Chierico et al., 2016; Yang et al., 2021). Meanwhile, other gut microbiota also plays important role in regulating hyperlipidemia, which demands further investigation. The results showed that the increase of several healthy microbial genera and the decrease of potentially pathogenic microorganisms were the main characteristics of FRRT in improving the disorder of gut microbiota.

Our results also showed that FRRT alleviated the high-fat diet-induced changes in amino acid metabolites (glutamine, glutamic acid, alanine, etc.), fatty acid metabolites (dodecanoic acid, myristic acid, decanoic acid, etc.), and bile acids metabolites (HDCA, LCA, DCA, etc.).

The deoxycholic and lithocholic acids of secondary bile acids were converted from primary bile acids by intestinal bacteria (Ridlon et al., 2005). Accumulating studies showed that a high-fat diet could lead to the increased production of secondary bile acids such as DCA and LCA, and evidence suggests that DCA and LCA are toxic bile acids associated with metabolic syndrome and gastrointestinal diseases (Bernstein et al., 2006; Stenman et al., 2012; Stenman et al., 2013). The farnesoid X receptor (FXR) is a bile acid (BA) receptor activated by specific BA metabolites, When FXR is activated, it inhibits the production of bile acids, inhibits the production of lipids, decreases triglycerides, and lowers blood glucose (Jiao et al., 2017; Sun et al., 2021; Xiang et al., 2021). Our studies showed that FRRT alleviated the fat-diet induced increases in fecal BAs such as DCA and LCA, which is consistent with the previous studies (Holscher et al., 2018; Wang et al., 2020; Yan et al., 2021). Our studies also showed that DCA, LCA was significantly positively correlated with TC, TG, LDL-c, and body weight and negatively correlated with the gut microbiota of *Prevotella*, *Oscillospira*, *Paraprevotellaceae_Prevotella*, *Ruminococcus*. These findings imply that FRRT may suppress FXR signaling in the liver and maintain homeostasis of BA enterohepatic circulation to improve dyslipidemia.

Meanwhile, the levels of amino acids in fecal metabolites were altered significantly. Some studies showed that amino acids play an important role in metabolism (Wu, 2009), which are mainly involved in various metabolic pathways, such as the tricarboxylic acid (TCA) cycle, gluconeogenesis, and others (Park and Han, 2018). l-alanine can affect *ß* cell signal transduction and apoptosis (Park and Han, 2018). High glutamine reduces glycolysis and inflammation in human fat cells and attenuates adipose tissue inflammation (Petrus et al., 2020), glutamic acid can be converted into aspartic acid through the TCA cycle, which plays an important role in glycolysis with energy metabolism (Davalli et al., 2012). Our studies showed that the contents of glutamic acid, glutamine, alanine, leucine, and isoleucine in the feces were increased in rats with FRRT treatment. The studies also showed amino acids such as glutamic acid, glutamine, alanine, leucine, and isoleucine that were significantly positively correlated with TC, TG, and gut microbiota of *Oscillospira*, *Prevotella*, *Para-prevotellaceae_Prevotella*, *Ruminococcus*. It implies that amino acids may accelerate the TCA cycle, leading to increased energy expenditure. However, the exact mechanisms are still too complex and need to be studied in more depth. In addition, some studies reported that fatty acids also play important roles in regulating energy metabolism. Such as myristic acid ameliorates hyperglycemia in genetic type 2 diabetes (Takato et al., 2017).

In this study, 16S rDNA sequencing and liquid mass spectrometry were used to study the changes in gut microbiota structure and metabolic function with FRRT treatment. In the high-fat diet-induced metabolic disorder syndrome of rats, gut microbiota composition and related metabolic pathways were significantly changed. In brief, FRRT mainly plays an important role in lowering blood lipid by affecting related bacteria (*Prevotella*, *Oscillospira*, *Paraprevotellaceae_Prevotella*, *Ruminococcus*) and their associated metabolites (amino acid metabolites, bile acid metabolites, and lipid metabolites).

FRRT significantly modified the structure and composition of gut bacteria and the levels of their related metabolites in rats. FRRT can prevent high-fat diet-induced hyperlipidemia, and mainly exerts a hypolipidemic effect by regulating the composition of gut microbiota and its metabolites. In addition, there was some limitation of the experiment that the lack of negative control, which makes it difficult to determine whether the values of the investigated parameters are close to those of healthy animals. Besides, the experiment only conducted a single-dose study and did not investigate the appropriate dose range and potential therapeutic relevance, which has limitations and incompleteness. Our study suggests that FRRT may serve as a new dietary and therapeutic strategy to improve dyslipidemia while also extending the understanding of the relationship between gut microbiota, metabolites, and lipid-lowering function. We need further studies to do so confirm the hypothesis (Deng et al., 2018; Jin et al., 2018).

## Data Availability

The datasets presented in this study can be found in online repositories. The names of the repository/repositories and accession number(s) can be found below: NCBI Sequence Read Archive (SRA) database under the Accession Number of SRP373909.
